# A SAXS/WAXD beamline with laboratory SAXS apparatus at NanoTerasu

**DOI:** 10.1107/S1600576725002547

**Published:** 2025-04-25

**Authors:** H. Meguro, M. Nishibori, H. Yamane, Y. Kotani, Y. Watanabe, K. Kamitani, T. Nakamura, K. Matsumura, M. Takata, Y. Takasaki, N. Yagi

**Affiliations:** a Photon Science Innovation Center, Sendai, Japan; b International Center for Synchrotron Radiation Innovation Smart Tohoku University Sendai Japan; c Ebara Corporation, Fujisawa-shi, Kanagawa, Japan; d Anton Paar Japan, Sumida-ku, Tokyo, Japan; NSRRC, Taiwan

**Keywords:** small-angle X-ray scattering, wide-angle X-ray diffraction, NanoTerasu, branch beamlines

## Abstract

NanoTerasu is a new 3 GeV synchrotron radiation facility in Sendai, Japan, which began user operation in April 2024. A station for small-angle X-ray scattering (SAXS) and wide-angle X-ray diffraction (WAXD) has been built at the wiggler beamline BL08W. The station was built in a branch for which an X-ray beam is provided by Bragg reflection from two Si crystals. The X-ray energy is 8.0 or 13.1 keV using either an Si(111) or an Si(220) crystal, respectively. The beam is focused with an Ru-coated bent cylindrical mirror. In the SAXS station, an Anton Paar SAXSpoint 5.0 laboratory X-ray apparatus has been installed. The original X-ray generator and focusing mirror in the laboratory equipment were removed and four-quadrant slits and an ion chamber have been installed. Low-scatter slits in SAXSpoint 5.0 are used as guard slits in front of the sample. The X-ray detector is a DECTRIS Eiger2 1M. The slits, sample and detector are enclosed in a vacuum chamber to reduce scattering from the air. The detector can be moved along the X-ray axis under vacuum to change the sample-to-detector distance between 53 and 1610 mm. The X-ray flux at 8.0 keV is 1.0 × 10^11^ cps with a ring current of 200 mA, shortening the exposure time by nearly 1000 times compared with the original laboratory X-ray source. The purpose of employing SAXSpoint 5.0 is to provide a user-friendly SAXS/WAXD system. Anton Paar’s measurement control system is employed in user operations. Many users utilize a multi-sample holder onto which 20 samples are fixed and measured in turn as programmed. The data analysis software is also provided by Anton Paar, but data can optionally be stored in TIFF format to be processed by any software that users are familiar with. Among the various sample attachments available from Anton Paar, a goniometer stage for grazing-incidence SAXS/WAXD is employed by many users.

## Abstract 2

Laboratory small-angle X-ray scattering (SAXS) equipment was employed at the SAXS station at NanoTerasu (Sendai, Japan) for SAXS and wide-angle X-ray diffraction (WAXD) measurements.

## 1. Introduction

NanoTerasu is a new 3 GeV synchrotron radiation facility in Sendai, Japan, which began user operation in April 2024. It is designed to be a low-emittance storage ring with a horizontal emittance of 1 nm rad. The circumference of the storage ring is 349 m. Some of the beamlines were built under the ‘Coalition Concept’, which is an industry–academic alliance for synchrotron radiation (SR) applications (Takata *et al.*, 2019 ▸). At these beamlines, particularly those with standard SR measurement capabilities, the main users are expected to be from industry and unskilled in the use of SR equipment. Before construction it had been recognized that beamlines for such users must be user-friendly.

Small-angle X-ray scattering (SAXS) beamlines at SR facilities are usually built to achieve the high performance that is necessary to carry out leading-edge experiments by users who are specialized in the technique. On the other hand, new users often find it difficult to carry out basic measurements with such advanced apparatus. As for data analysis, even though software is developed at most SAXS beamlines, there is still no standard, user-friendly software even for circular averaging or calibration of camera length. Currently these are significant barriers for users who want to measure their samples at SAXS beamlines for the first time.

In parallel with the advent of synchrotron facilities, there has been notable progress in the performance of laboratory systems for SAXS/wide-angle X-ray diffraction (WAXD) measurements such as Xeuss 3.0 from Xenocs (https://www.xenocs.com/saxs-products/saxs-equipment-xeuss/), NANOSTAR from Bruker (Lyngsø & Pedersen, 2021 ▸) and SAXSpoint 5.0 from Anton Paar (https://www.anton-paar.com/us-en/products/group/saxs-instrumentation/). Despite lower X-ray flux, these systems have advantages over SAXS beamlines at SR facilities. (1) A variety of sample environments are available and integrated in the measurement software; users can also readily purchase sample holders for a specific measurement from the manufacturer. (2) Built-in user-friendly measurement software is provided. (3) Data analysis software, integrated with the measurement system, is available. In these systems, the optics, sample environment and detector are under vacuum to eliminate air-scattering and also to facilitate changing the sample-to-detector distance (SDD). Use of a photon-counting detector is also an attractive feature.

Considering these merits, the Anton Paar SAXSpoint 5.0 laboratory SAXS apparatus was introduced at the SAXS endstation at NanoTerasu. However, since it was not designed for a synchrotron light source, modifications had to be made to achieve satisfactory performance, as described in this paper. The all-vacuum design of SAXSpoint5.0 not only lowers the background in data but also facilitates changing the SDD, which is often hard work at SAXS beamlines. The integrated software provided by Anton Paar enables automatic measurement of samples and allows a smooth workflow from measurement to analysis, which is ideal for non-specialist users.

## 2. Beamline description

**Figure 1:**
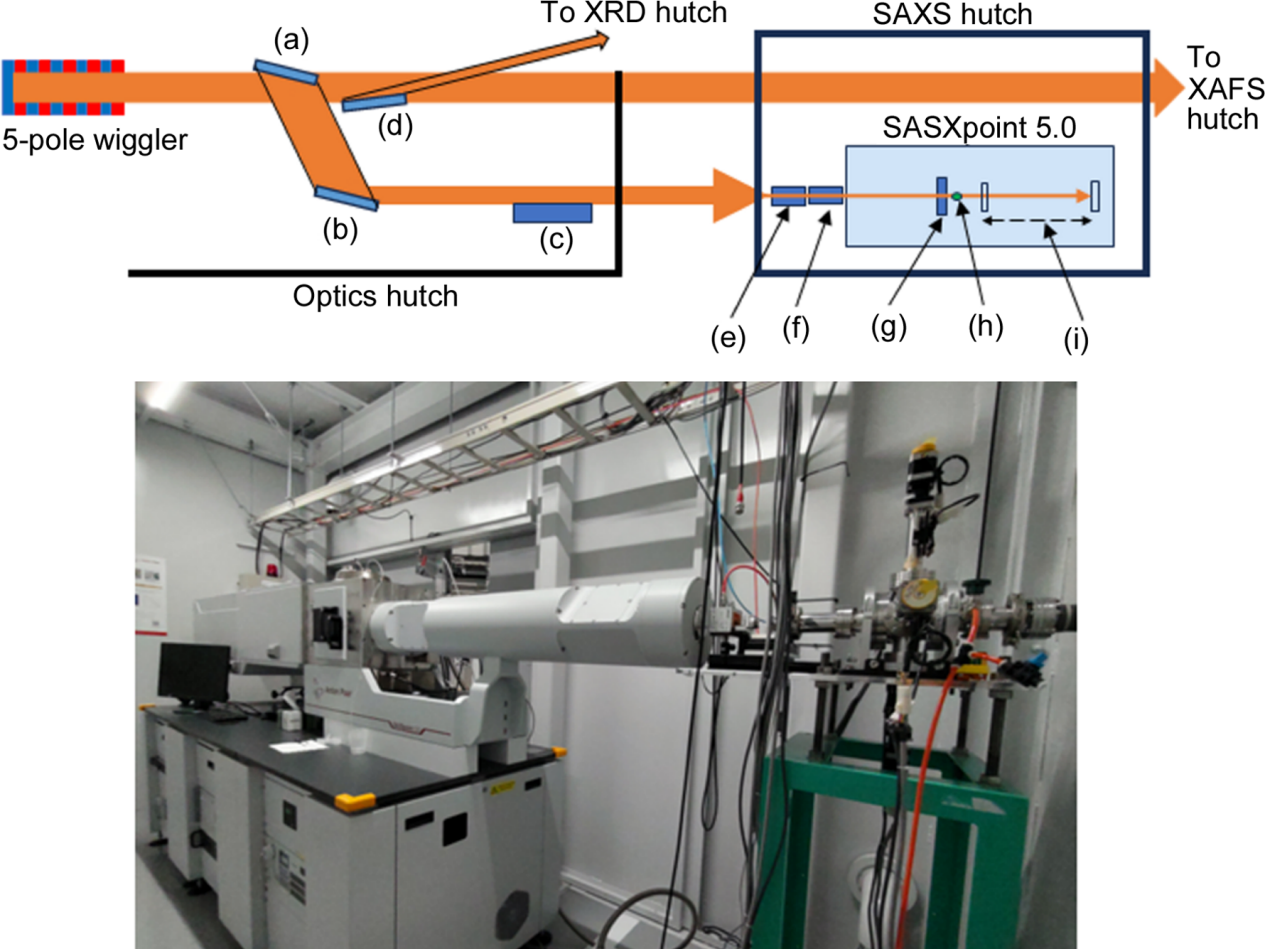
(Top) Schematic of the BL08W SAXS branch at NanoTerasu. (a) First branch crystal. (b) Second branch crystal. (c) Focusing mirror. (d) Branch crystal for the XRD endstation. (e) Four-quadrant slits. (f) X-ray shutter, absorber and ion chamber. (g) Low-scatter slits. (h) Sample position. (i) Eiger2 1M with a beamstop. (Bottom) Photograph of the apparatus in the hutch.

The SAXS/WAXD station was built at the 5-pole wiggler beamline BL08W in NanoTerasu. In order to accommodate as many endstations as possible, an X-ray beam with a horizontal divergence of 1.0 mrad is split into three sections by Bragg reflection from Si crystals in the horizontal plane (Fig. 1 ▸, top). In the case of the SAXS station, an Si(111) or Si(220) crystal with a Bragg angle of 14.25° is used to reflect up to 0.05 mrad from the edge of the beam in the horizontal plane. The reflected beam is further reflected by an Si(111) or Si(220) crystal to align the beam parallel to the original beam path. The surfaces of the two crystals are vertical, so that by shifting them along the vertical axis, X-rays of either 8.0 or 13.1 keV can be used in the endstation. This technique of beam splitting will be described in a future paper. The X-ray beam is reflected by an Ru-coated bent cylindrical Si mirror with a glancing angle of 4.2 mrad to remove harmonic reflections and to focus the beam at the sample position. The specifications of the beamline are summarized in Table 1 ▸.

The upstream wall of the SAXS hutch is 35.6 m from the X-ray source. The dimensions of the hutch are 5 m (length along the beam), 4 m (width) and 3 m (height). SAXSpoint 5.0 from Anton Paar, which can fit into a hutch of this size, has been installed (Fig. 1 ▸, bottom). Although SAXSpoint 5.0 for laboratory use is equipped with a micro-focus X-ray source and focusing mirror, these were removed for use with synchrotron X-rays. In the hutch, a four-quadrant slit system (Kohzu, Japan), a solenoid-based X-ray shutter, a remote-controlled absorber system and an ion chamber (S-1329A1, OKEN, Japan) were installed on an optical bench upstream of SAXSpoint 5.0. The main part of SAXSpoint 5.0 was elevated by 150 mm with spacers to adjust the height of the beam (1.4 m).

In SAXSpoint 5.0 there are two sets of low-scatter slits before the sample stage. However, to increase the distance between slit systems, the four-quadrant slit upstream of SAXSpoint 5.0 was installed. The rear slits in SAXSpoint 5.0 are close to the sample position and work as guard slits. The distance from these slits to the sample varies from about 50 to 200 mm depending on the translation of the sample stage.

Originally there was an X-ray shutter associated with the X-ray source. As this was removed, a fast solenoid-driven X-ray shutter was required to avoid unnecessary irradiation on the sample. The shutter opens in 1 ms with a delay of 13 ms after a trigger pulse, and closes in 2 ms with a delay of 20 ms. In addition to the absorbers upstream, there is a set of absorbers in SAXSpoint 5.0 that can be changed remotely.

The X-ray detector is an Eiger2 1M (DECTRIS, Switzerland) which is on a bench that can be moved along the beam. By also moving the sample stage, the longest SDD is 1610 mm while the shortest is 53 mm. There are three beamstops – 1 mm W, 2 mm or 3 mm Ni – on a rotary base so that they can be selected by rotation. They are located just in front of the detector and move together with the detector along the bench. The beamstop is placed at the bottom of the detector so that a wider range of scattering vector can be measured. The detector can be translated both vertically and horizontally perpendicular to the beam, but care must be taken not to let the intense X-ray beam fall on the detector because the beamstop moves together with the detector.

In SAXSpoint 5.0, the slits, absorber, sample and detector are enclosed in a vacuum chamber to eliminate scattering by air. This also enables translation of the detector along the bench by remote control, facilitating the change in SDD. It takes about 8 min to reach a vacuum level of <100 Pa.

Currently, only the original control software provided by Anton Paar is used despite the fact that we have changed the X-ray source from an internal to an SR source. Since the software assumes the use of an internal X-ray source, it does not take into account the high flux of SR X-rays. For example, X-ray transmittance of a sample is measured by the Eiger detector without a beamstop. For this measurement, the software sets the absorber to the low position, but the intense SR X-rays can saturate and damage the detector. The users have to change the absorber from low to high manually. Software changes to cope with the different X-ray energy and the high flux are being discussed with Anton Paar and will be realized in future.

## 3. Performance of the system

The 8.0 keV X-ray flux measured by the ion chamber was 1.0 × 10^11^ photons s^−1^ with a ring current of 200 mA, shortening the exposure time by three orders of magnitude compared with the original laboratory X-ray source. At 13.1 keV, the flux was 4.4 × 10^10^ photons s^−1^.

**Figure 2:**
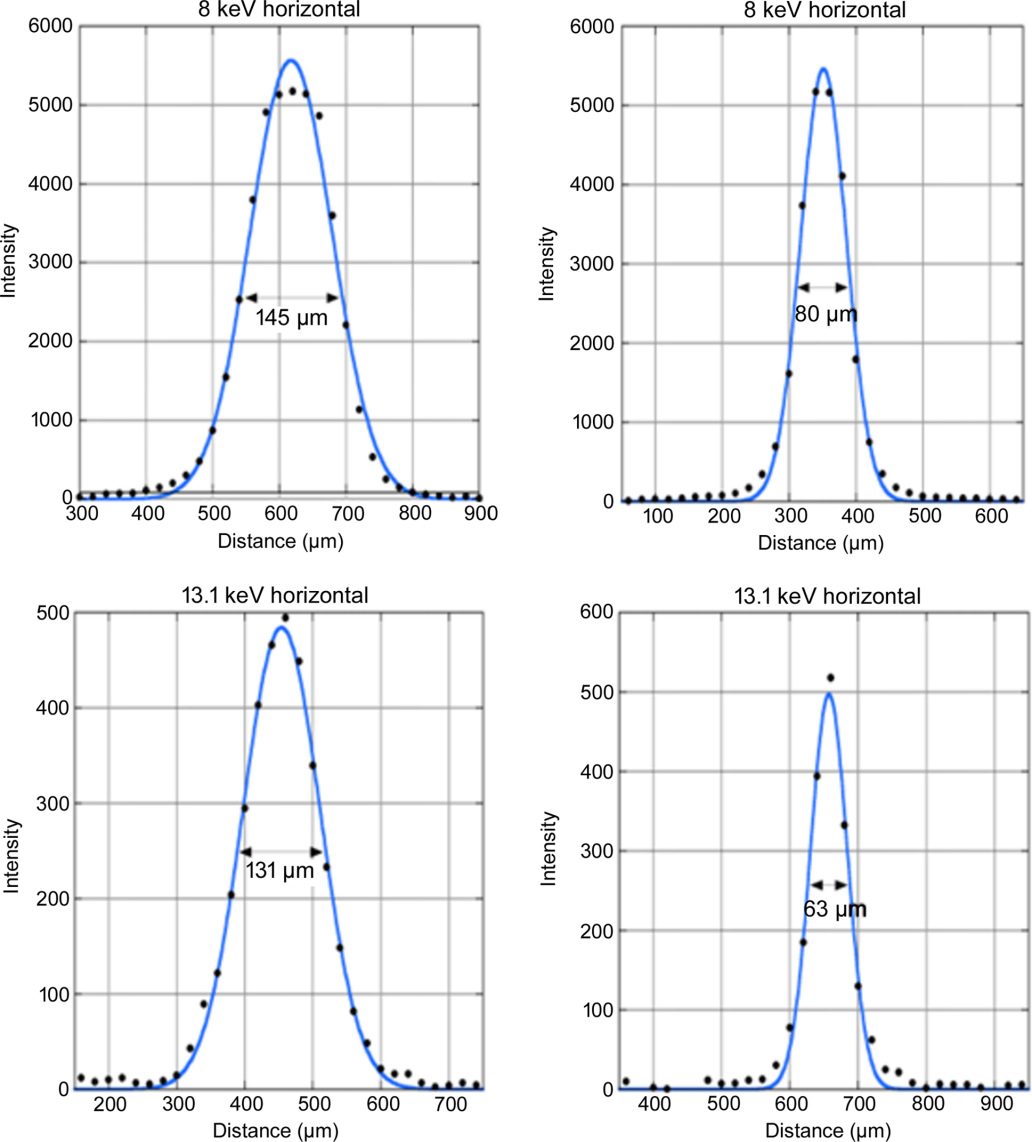
Beam profiles at the sample position with X-ray energies of 8.0 and 13.1 keV. The data (dots) were fitted with a Gaussian function (blue curve).

The beam size was measured with a small flat-panel detector for dental use (S11684, Hamamatsu Photonics, Japan) with a pixel size of 20 µm. At the sample position the FWHM was 145 µm in width and 80 µm in height at 8.0 keV, and 131 µm in width and 63 µm in height at 13.1 keV (Fig. 2 ▸).

**Figure 3:**
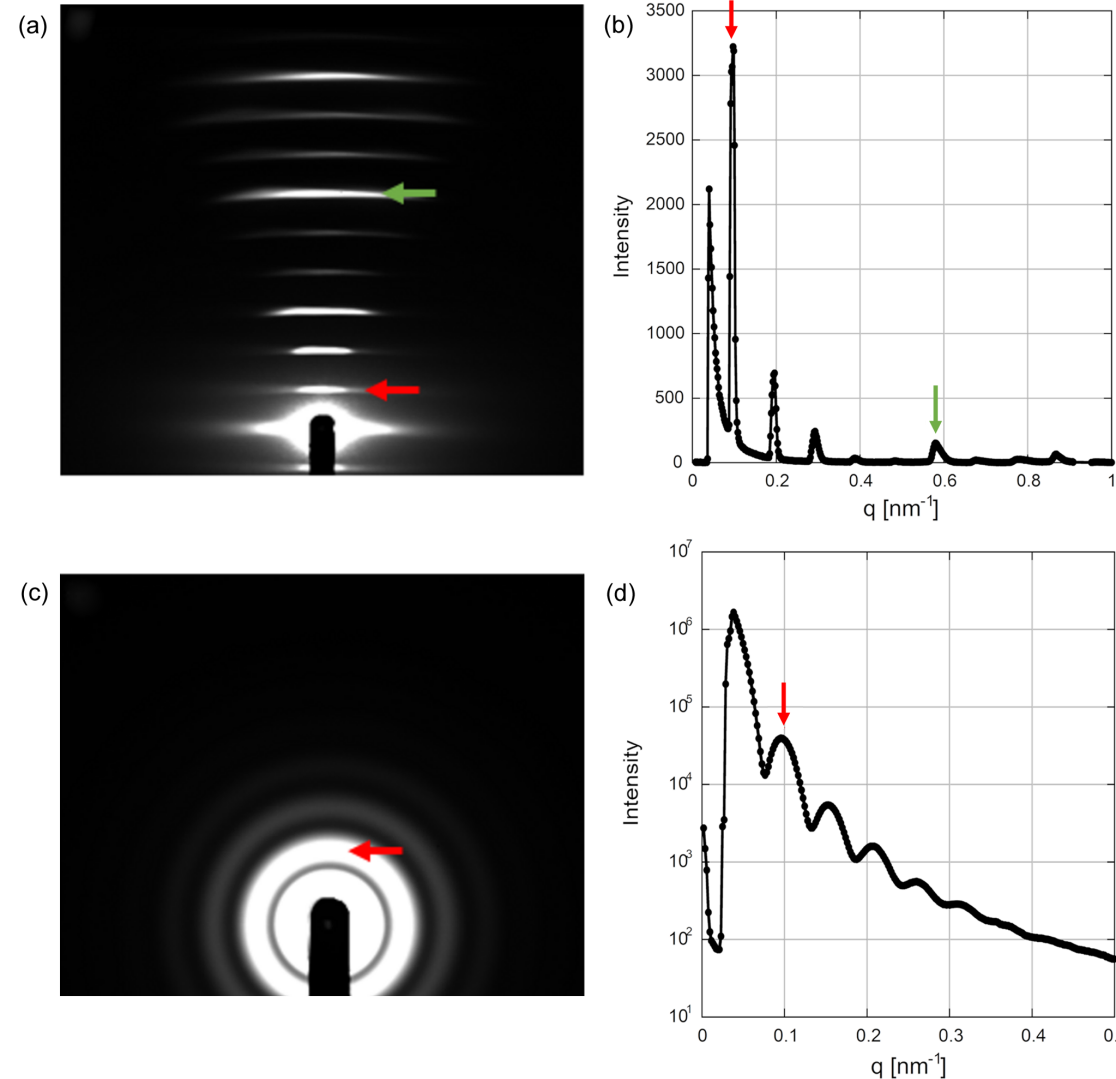
Examples of data acquired with an X-ray energy of 8.0 keV. (*a*) Small-angle diffraction from dry rat tail tendon collagen. The first peak (red arrow) is at *d* = 63 nm while the green arrow is the sixth order at *d* = 10.5 nm^−1^. The exposure time was 5 s. The SDD was 1607 mm. (*b*) Vertical (meridional) intensity profile of (*a*). The red and green arrows indicate the first and sixth meridional reflections. (*c*) Scattering from a *ca* 0.5% solution of silica particles with a nominal diameter of 100 nm (Seahoster KE-W10, Nippon Shokubai, Japan) in a 1.5 mm capillary. The exposure time was 60 s. The SDD was 1600 mm. The red arrow indicates the first peak at *q* = 0.096 nm^−1^. (*d*) Circular-averaged scattering curve of (*c*). The red arrow indicates the first peak.

Table 2 ▸ shows the minimum and maximum scattering vector magnitudes (*q* = 4π sin θ / λ, where 2θ is the scattering angle) measurable at this beamline. The minimum *q* values are calculated at the edge of the 2 mm-wide Ni beamstop with the beam at its center. In the calculation, the minimum *q* at the longest SDD is 0.025 nm^−1^, corresponding to a *d*-spacing of 250 nm. However, in practice, central scattering from the optics limits the largest *d*-spacing to around 100 nm. Fig. 3 ▸ shows typical scattering and diffraction results using this system. The diffraction from collagen [Figs. 3 ▸(*a*) and 3 ▸(*b*)] shows the first-order reflection from the *D* period (63 nm in dry rat tendon), which is the axial stagger between neighboring collagen molecules (Worthington & Tomlin, 1955 ▸; Petruska & Hodge, 1964 ▸). Figs. 3 ▸(*c*) and 3 ▸(*d*) show scattering from a solution of silica particles with a nominal diameter of 100 nm with the first peak at *q* = 0.096 nm^−1^. Since the first peak from a sphere is expected at *qR* = 5.8, where *R* is the radius, the actual radius is estimated to be 121 nm.

**Figure 4:**
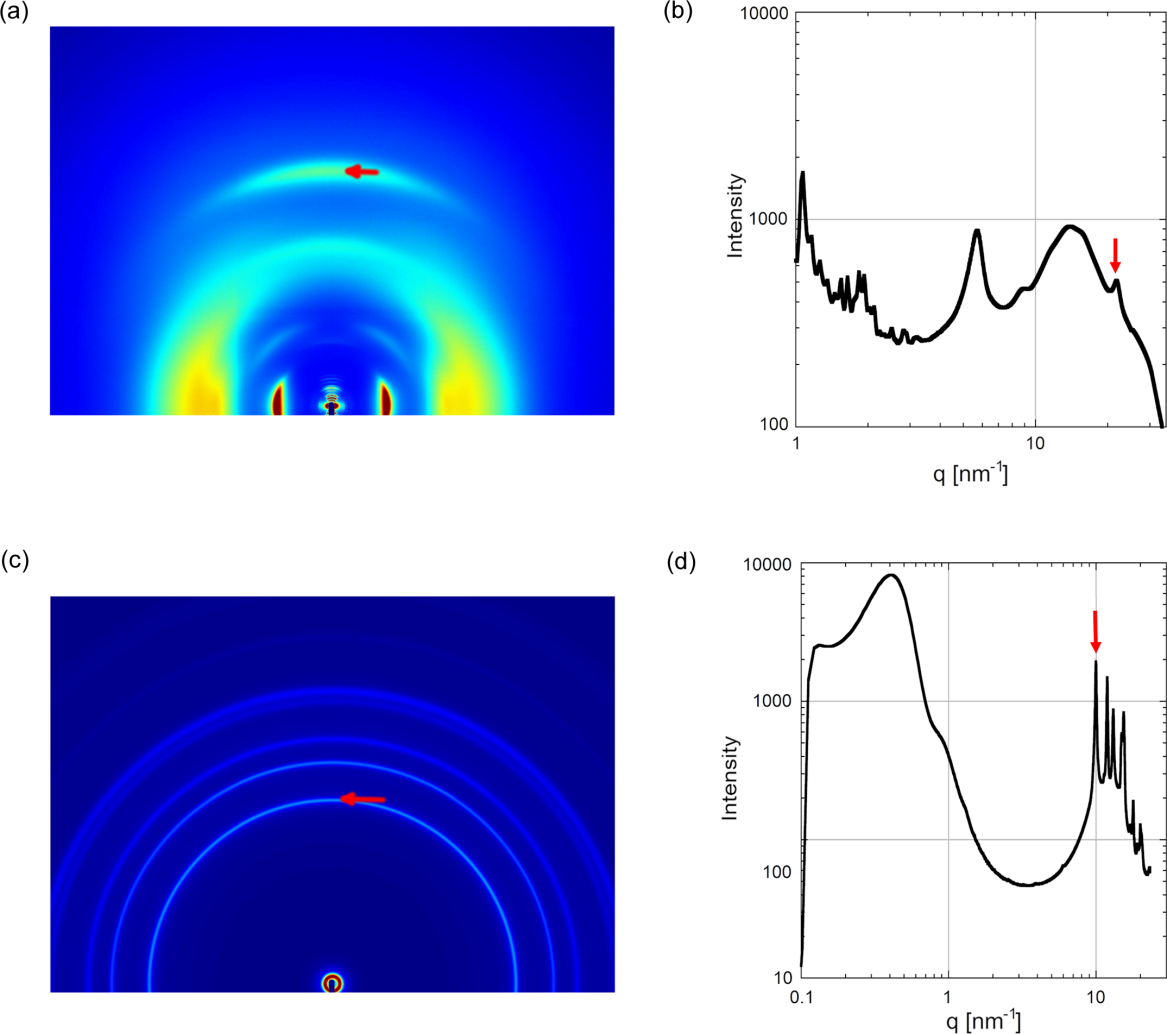
Examples of data acquired with an X-ray energy of 8.0 keV. (*a*) Extended diffraction pattern from dry rat tail tendon collagen. Six diffraction patterns at different detector positions, each of which was made of two patterns to fill the gap between detector modules, were combined. The exposure time was 60 s. SDD = 160 mm. The red arrow indicates the meridional reflection at *d* = 0.286 nm. (*b*) Circular-averaged intensity profile of (*a*). The red arrow indicates the same peak as in (*a*). (*c*) Extended diffraction pattern from polypropyl­ene (FY4). The exposure time was 10 s. SDD = 300 mm. The green arrow at *q* = 0.40 nm^−1^ indicates the peak from a lamellar structure, while the red arrow at *q* = 9.99 nm^−1^ indicates a peak from the crystalline structure of polypropyl­ene. (*d*) Circular-averaged scattering curve of (*c*). The two arrows indicate the lamellar peak (green) and the crystalline peak (red).

Although Eiger2 1M is a small detector, the *q* range can be expanded by moving the detector in the vertical and horizontal directions and combining the acquired images (Table 2 ▸). The gap between the modules in the detector is also covered by taking two exposures with a vertical shift of the detector. The software provided by Anton Paar (*SAXSdrive* and *SAXSanalysis*) can be used to acquire multiple images and combine them. Although these protocols take multiple exposures, the short exposure time makes such operations practical thanks to the high flux. Fig. 4 ▸(*a*) is a diffraction pattern from rat collagen showing orders of reflections from the large *d*-spacing (63 nm) in the small-angle region and wide-angle reflections arising from the arrangements of fibrils and amino acid residues (Rich & Crick, 1961 ▸) [Fig. 4 ▸(*b*)].

Figs. 4 ▸(*c*) and 4 ▸(*d*) demonstrate the possibility of simultaneous SAXS/WAXD measurements with the small detector. From a polypropyl­ene sample, the lamellar peak at about *q* = 0.4 nm^−1^ is observed together with the crystalline peaks beyond 6 nm^−1^.

**Figure 5:**
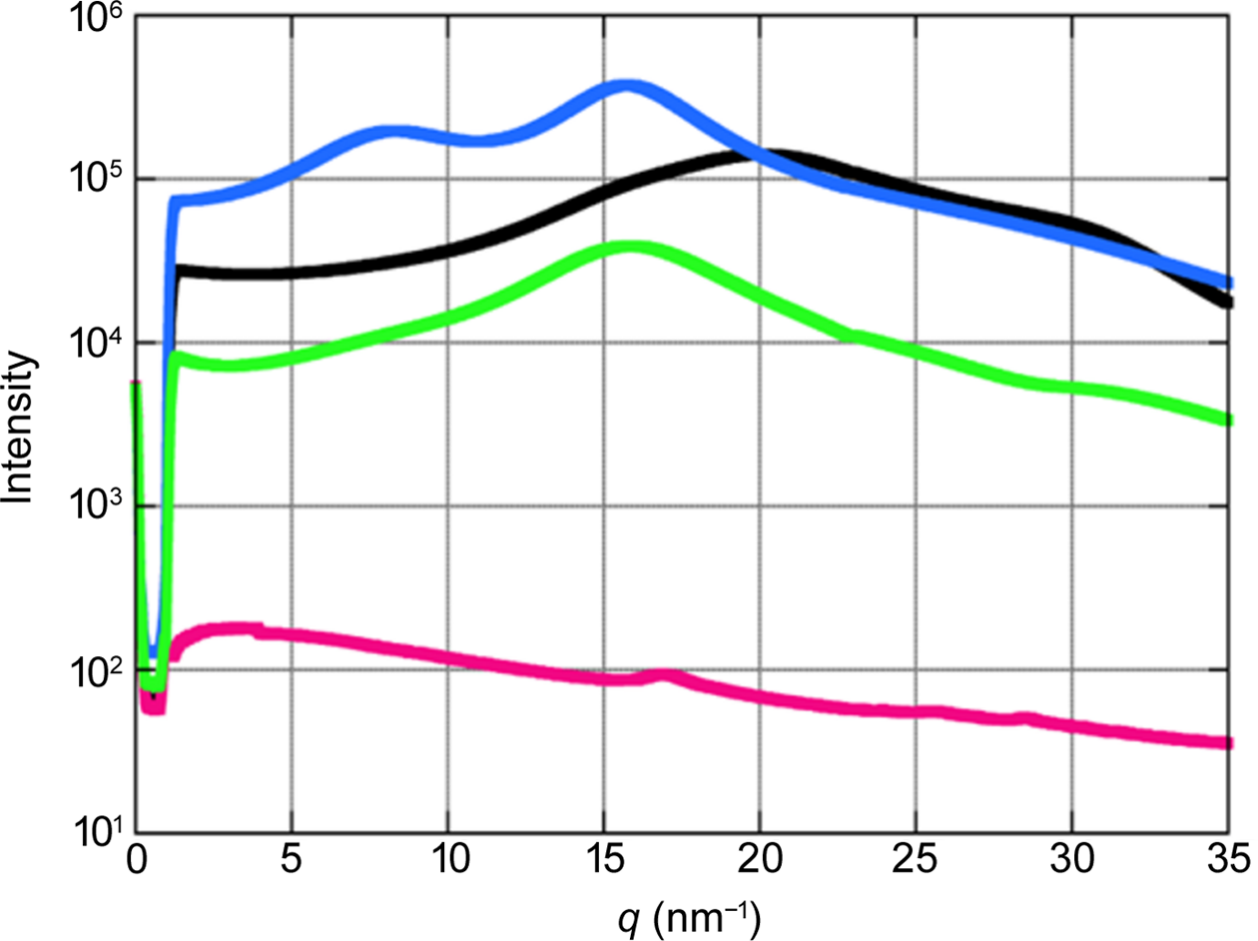
Circular-averaged scattering curves from water (black), ethanol (blue), an empty capillary tube (green) and without a capillary tube (red). The samples were sealed in 1.0 mm glass (borosilicate) capillary tubes with a wall thickness of 10 µm. The exposure time was 60 s. SDD = 60 mm. The X-ray energy was 8.0 keV. The small peak at about *q* = 16 nm^−1^ in the blank exposure (red) is due to parasitic scattering from the capillary holder.

Fig. 5 ▸ shows scattering of water and ethanol in a glass capillary, demonstrating the low background owing to the vacuum environment. The peaks in the scattering from water (black curve) and ethanol (blue curve) indicate the presence of a molecular network structure, allowing analysis of the pair distribution function (Nilsson *et al.*, 2016 ▸; Pethes *et al.*, 2020 ▸). Absence of air scattering helps the data analysis, but care must be taken to remove scattering from the sample cell, which is a glass capillary in Fig. 5 ▸.

## 4. Standard sample environments

The purpose of employing SAXSpoint 5.0 at this beamline is to provide a user-friendly SAXS/WAXD system. Anton Paar’s measurement control system *SAXSdrive* is employed by the users. The SDD can be changed by simply inserting a value in the software between 53 and 1610 mm. The sample stage has remotely controlled horizontal and vertical movements. The standard sample holder is a solid 5 × 4 holder onto which 20 samples can be set and measured in turn as programmed. Since this holder is available from Anton Paar, users can have their own holders and set samples on them in their laboratory before coming to the beamline. With the standard temperature control unit (heated/cooled sampler), the sample temperature can be changed between −10 and 120°C. The vacuum facilitates measurements at low temperature because condensation does not occur. With the optional Heated Module 2.0, the range is from ambient to 350°C. The standard data format is compressed HDF5, but data can also be stored in TIFF format to be processed by any software that users are familiar with. The data analysis software provided by Anton Paar (*SAXSanalysis*) can be used not only for circular averaging of centrosymmetric scattering but for a variety of data analysis methods such as Guinier analysis and Porod analysis. A dataset acquired by *SAXSdrive*, for example scattering patterns from 20 samples on the 5 × 4 holder, can be processed by *SAXSanalysis* collectively. This helps to accelerate data analysis so that users can examine data acquired during measurements. Such integration is one of the advantages of using a commercial integrated control/analysis system.

Grazing-incidence (GI) SAXS and WAXD can be performed with a goniometer from Anton Paar. With this system, the shortest SDD is 80 mm. The glancing angle can be changed with a precision of 0.0001°. Rotation of the sample around the vertical axis is also possible to enable measurements of molecular orientation in the sample.

Although the vacuum environment is ideal to eliminate air scattering, there are samples such as liquids that cannot be measured under vacuum. These samples can be enclosed in capillaries (Fig. 5 ▸). Another option is a flow cell with which sample solution can be flowed through tubing to a quartz capillary tube from outside of the vacuum. A drawback is that this requires a larger amount of solution, at least a few tens of microlitres. These techniques are currently available at the beamline. Using this cell and flow system, it is possible to perform continuous measurements of proteins eluted from a column (SEC–SAXS).

Automatic measurements with an autosampler and multimodal UV/Vis or FTIR measurements of liquid samples are also possible with optional apparatuses from Anton Paar. Other attachments such as a high-pressure cell, cryo-module (down to −150°C), rheoSAXS module, shear cell, tensile stage and humidity stage are also available from Anton Paar. These attachments will be purchased in future according to the requirements of the users.

Although ready-made sample environments provided by Anton Paar are useful and user-friendly, some samples do not fit the holders. Also, under vacuum, experiments that cannot be performed with a ready-made apparatus are difficult. Such experiments require an air-filled box with X-ray transparent windows to enclose the sample, which has to be prepared by the users according to the requirements of their experiments. To facilitate user-designed experiments, feed-through adapters and connectors for gas, ethernet, cooling water and liquid (PEEK tube) are available between air and vacuum. There is a free port to mount adapters that are designed and prepared by users.

An example that circumvents difficulties of in-vacuum measurement is the Anton Paar heating module for the GI-SAXS stage with two Kapton windows in which a sample can be kept in air. With this stage, it is possible to perform GI-SAXS at temperatures up to 500°C. There is a gas inlet and outlet connected to out-of-vacuum for a continuous flow of an inert gas. This can be also used to release a gas that emerges from the sample by heating. Structural changes in a thin polymer film at high temperature can be studied using this equipment.

## 5. Conclusions

A design incorporating a large vacuum vessel in order to allow an X-ray detector to be translated along the beam path has been employed at several synchrotron SAXS beamlines such as ID2 at ESRF (Narayanan *et al.*, 2022 ▸), SWING at Soleil (https://wwwpreprod.synchrotron-soleil.fr/en/beamlines/SWING), CoSAXS at MAX IV (Kahnt *et al.*, 2021 ▸), BioSAXS at ANSTO (https://www.ansto.gov.au/biological-small-angle-xray-scattering-beamline) and 13A at TPS (Shih *et al.*, 2022 ▸). In all cases, a large vacuum tube was constructed to enclose a large X-ray detector. In SAXSpoint 5.0, since a smaller detector (Eiger2 1M) is used, the vacuum system is compact. This design is suitable for a small endstation. A larger *q* range can be covered by moving the detector and combining multiple scattering patterns. Its low price is also attractive, costing approximately less than a quarter of the price of a large SAXS beamline. The all-vacuum SAXS system ensures low-background data. However, compared with a system in which the sample is in the air, there are disadvantages that have to be overcome with adequate apparatus.

The sample environments provided by Anton Paar are designed for standard measurements rather than the complicated experiments envisioned by some users. Generally, compared with SAXS beamlines in SR facilities, there are limitations in the flexibility of laboratory SAXS systems. On the other hand, a user-friendly system is realized owing to these limitations. Where there is more emphasis on user friendliness than flexibility, use of a laboratory SAXS system can be an appropriate choice, as demonstrated at this beamline. From the viewpoint of beamline management, it is also notable that maintenance and troubleshooting can be taken care of by service engineers from the manufacturer. This greatly reduces the workload of the beamline staff.

## Figures and Tables

**Table 1:** Specifications of the BL08W SAXS beamline

X-ray source	5-pole wiggler
Monochromator	Two Si crystals [(111) or (220)]
X-ray energy (keV)	8.0 or 13.1
Focusing mirror	Bent cylindrical Si mirror
SDD (mm)	53 (shortest) to 1610 (longest)

**Table:** Minimum scattering vector; the minimum *q* value is calculated at the edge of a 2 mm beam stop.

X-ray energy (keV)		8.0		13.1	
X-ray flux with 200 mA ring current (cps)		1.0 × 10^11^		4.4 × 10^10^	
Minimum *q* with the longest SDD (1610 mm) (nm^−1^)		0.025		0.041	
Minimum *q* with the shortest SDD (53 mm) (nm^−1^)		0.77		1.26	

**Table:** Maximum scattering vector; the maximum *q* value is calculated for the beam at the bottom center of the Eiger detector. For the single image, ‘top’ indicates *q* at the top center of the detector, while ‘corner’ means *q* at the top left or right corner of the detector. In the combined image, an image made by connecting six images is treated as a single image.

X-ray energy (keV)		8.0		13.1	
SDD (mm)		53	1610	53	1610
Single image	Maximum *q* at the top (nm^−1^)	38.1	2.00	62.9	3.28
	Maximum *q* at the corner (nm^−1^)	40.1	2.23	65.6	3.65
Merged image (6 positions)	Maximum *q* at the top (nm^−1^)	47.5	4.00	77.8	6.55
	Maximum *q* at the corner (nm^−1^)	49.4	4.93	80.9	8.07
